# Pyrotinib plus capecitabine for trastuzumab-resistant, HER2-positive advanced breast cancer (PICTURE): a single-arm, multicenter phase 2 trial

**DOI:** 10.1186/s12916-023-02999-0

**Published:** 2023-08-09

**Authors:** Jun Cao, Yuee Teng, Huiping Li, Lili Zhang, Quchang Ouyang, Weimin Xie, Yueyin Pan, Zhenchuan Song, Xiaoling Ling, Xiaohong Wu, Jingwei Xu, Li Li, Liping Ren, Hong Wang, Dongxian Zhou, Jing Luo, Xichun Hu

**Affiliations:** 1 Department of Breast and Urologic Medical Oncology, Fudan University Shanghai Cancer Center, 270 Dong’An Road, Shanghai, 200032 China; 2 Department of Medical Oncology, The First Hospital of China Medical University, 110001 Shenyang, China; 3 Department of Medical Oncology, Peking University Cancer Hospital and Institute, Beijing, 100142 China; 4 Department of Medical Oncology, Jiangsu Cancer Hospital, Nanjing, 210008 China; 5 Department of Medical Oncology, Hunan Cancer Hospital, Changsha, 410013 China; 6 Department of Medical Oncology, Guangxi Medical University Cancer Hospital, Nanning, 530027 China; 7 Department of Medical Oncology, The First Affiliated Hospital of USTC, Hefei, 230001 China; 8 Breast Center, The Fourth Hospital of Hebei Medical University, 050011 Shijiazhuang, China; 9 Department of Medical Oncology, The First Hospital of Lanzhou University, Lanzhou, 730013 China; 10 Department of Medical Oncology, Affiliated Hospital of Jiangnan University, Wuxi, 214122 China; 11 Department of Breast Surgery, The Second Hospital of Jilin University, Changchun, 130041 China; 12 Department of Medical Oncology, Qilu Hospital of Shandong University, Jinan, 250012 China; 13 Department of Breast Surgery, Guangdong Provincial Hospital of Chinese Medicine, Guangzhou, 510120 China; 14 Department of Medical Oncology, The Third Hospital of Nanchang, Nanchang, 330008 China; 15 Department of Breast Surgery, Shenzhen People’s Hospital, Shenzhen, 518020 China; 16 Department of Breast Surgery, Sichuan Provincial People’s Hospital, Chengdu, 610072 China

**Keywords:** Pyrotinib, Capecitabine, Trastuzumab resistance, Human epidermal growth factor receptor 2, Breast cancer

## Abstract

**Background:**

Patients with human epidermal growth factor receptor 2 (HER2)-positive advanced breast cancer and primary resistance to trastuzumab have a poor clinical outcome and lack good evidence to inform clinical decision. This study investigated the efficacy and safety of pyrotinib plus capecitabine in this population.

**Methods:**

This phase 2 trial was conducted at 16 sites in China. Patients received oral pyrotinib 400 mg once daily and capecitabine 1000 mg/m^2^ twice a day on days 1–14 of each 21-day cycle until disease progression or intolerable toxicity. The primary endpoint was investigator-assessed progression-free survival (PFS).

**Results:**

Between June 2019 and September 2021, 100 patients were enrolled with a median age of 51 years (range, 24–69). All patients had been treated with trastuzumab and 21 (21.0%) patients had prior use of pertuzumab. As of August 31, 2022, the median follow-up duration was 20.1 months (range, 1.3–38.2). The median PFS was 11.8 months (95% confidence interval [CI], 8.4–15.1), which crossed the pre-specified efficacy boundary of 8.0 months. The objective response rate was 70.0% (70/100), with a median duration of response of 13.8 months (95% CI, 10.2–19.3). The disease control rate was 87.0% (87/100). The median overall survival was not reached. The most common grade ≥ 3 treatment-emergent adverse event was diarrhea (24 [24.0%]). No treatment-related deaths occurred.

**Conclusions:**

Pyrotinib plus capecitabine can be considered to be a treatment option in HER2-positive advanced breast cancer patients who have shown primary resistance to trastuzumab. Even in the era of modern anti-HER2 treatments, this clinical setting warrants more investigations to meet unmet needs.

**Trial registration:**

ClinicalTrials.gov, NCT04001621. Retrospectively registered on June 28, 2019.

**Supplementary Information:**

The online version contains supplementary material available at 10.1186/s12916-023-02999-0.

## Background

Human epidermal growth factor receptor 2 (HER2)-positive breast cancer is an aggressive disease subtype, which accounts for 15–20% of breast cancers [1]. Trastuzumab is the first approved targeted biological, which significantly changes the natural course of this disease. In exposure to trastuzumab, 14–31% of early breast cancer and nearly all of advanced breast cancer can develop primary or secondary resistance to trastuzumab [2–8]. Those primary-resistant patients have either early relapse in the early setting or early progression in the advanced setting, representing approximately 30–50% of total recurrence [2–6] and approximately 1–25% of total progression [7, 8]. Patients with primary trastuzumab resistance are likely to be more aggressive and might derive no benefit from rechallenge of trastuzumab [9], while almost all recruited patients in the early-stopped phase 3 GBG26/BIG 3–05 trial were secondarily resistant to trastuzumab and could really benefit from it [10]. Therefore, there are unmet needs for patients with primary trastuzumab resistance.

Definition of primary trastuzumab resistance is derived from and serves for clinical trials, while its value of guiding clinical practice is not clarified. The cutoff to differentiate primary resistance from secondary resistance is not completely consistent in different clinical trials [11–18]. Previous clinical trials which only enrolled primarily trastuzumab-resistant, HER2-positive advanced breast cancer added the mammalian target of rapamycin (mTOR) inhibitor [11–13], tyrosine kinase inhibitor (TKI) [14], phosphatidylinositol 3-kinase (PI3K) inhibitor [15–17], or programmed cell death-1 inhibitor [18] to anti-HER2 therapy with or without chemotherapy, but all showed disappointing clinical benefits. Two phase 3 trials showed that patients with primary trastuzumab resistance had a median progression-free survival (PFS) of only 5.5 months with afatinib plus vinorelbine and 7.0 months with everolimus plus trastuzumab and vinorelbine, respectively [13, 14]. These PFS results were significantly shorter than others reported in patients with HER2-positive advanced breast cancer in a similar second-line setting, suggesting the aggressiveness of primary trastuzumab resistance and needing more investigations. Currently, the standard second-line treatment for HER2-positive advanced breast cancer has shifted from trastuzumab emtansine (T-DM1) to trastuzumab deruxtecan (T-DXd). However, the enrolled patients in the phase 3 trials of these antibody–drug conjugates were mixed population and patients with primary trastuzumab resistance were under-represented [19, 20]. It remains to be elucidated whether the primarily trastuzumab-resistant patients can derive the same benefit from novel anti-HER2 agents as others [19, 20].

Primary resistance mostly stands for intrinsic resistance and HER2 independency, while secondary resistance reflects enquired loss of sensitivity or presence of dominant resistant subclones [21]. Possible mechanisms of primary trastuzumab resistance include impaired binding to the extracellular domain of HER2, such as MUC4 or MUC1 expression [22, 23]; high expression of HER2 carboxy-terminal fragment p95HER2 [24]; HER2△16 lacking exon 16 [25]; activation of alternative signaling pathways, such as PI3K/protein kinase B (AKT)/mTOR and mitogen-activated protein kinase (MAPK) pathways [26, 27]; overexpression of insulin-like growth factor-1 receptor [28]; and epidermal growth factor receptor (EGFR) or HER3 amplification [29, 30]. Some newly investigated mechanisms include induction of immune suppression, vascular mimicry, breast cancer stem cells, and metabolic escape [31]. It is indicated that P95HER2, a truncated form of HER2 that lacks the extracellular domain to bind trastuzumab, is responsive to lapatinib [32, 33]. Pan-HER TKIs can also hinder tumor development through targeting other receptors (such as EGFR) that increase with trastuzumab exposure [34]. Thus, primary trastuzumab resistance might be overcome by pan-HER TKIs [32–35].

Pyrotinib is an irreversible pan-HER TKI that targets EGFR, HER2, and HER4. The phase 3 PHOEBE study confirmed the superiority of pyrotinib plus capecitabine over lapatinib plus capecitabine in patients with trastuzumab-taxane-treated, HER2-positive metastatic breast cancer [36]. Here we designed this phase 2 PICTURE study to investigate the efficacy and safety of pyrotinib plus capecitabine in patients with HER2-positive advanced breast cancer and primary trastuzumab resistance.

## Methods

### Study design and participants

This was an investigator-initiated, single-arm, phase 2 trial conducted at 16 sites in China. Patients were included if they were females aged 18–70 years; had pathologically confirmed HER2-positive (score 3 + by immunohistochemistry, or 2 + with positive results of fluorescence in-situ hybridization) locally advanced or metastatic breast cancer; had primary resistance to trastuzumab; had an Eastern Cooperative Oncology Group performance status of 0 or 1; had known hormone receptor status; had an expected survival of ≥ 3 months; had at least one measurable lesion according to the Response Evaluation Criteria In Solid Tumors (RECIST), version 1.1 [37]; and had adequate bone marrow (neutrophil count ≥ 1.5 × 10^9^/L; platelet count ≥ 100 × 10^9^/L; hemoglobin ≥ 90 g/L), hepatic (total bilirubin ≤ 1.5 × upper limit of normal [ULN]; alanine aminotransferase and aspartate aminotransferase ≤ 3 × ULN [≤ 5 × ULN for patients with liver metastases]), renal (creatinine ≤ 1.5 × ULN; creatinine clearance rate ≥ 50 mL/min), and cardiac (left ventricular ejection fraction ≥ 50%; Fridericia’s corrected QT interval < 480 ms) function. Based on the definitions used in previous clinical trials [14], primary trastuzumab resistance was defined as progression during (neo)adjuvant trastuzumab (subgroup A) or within 12 months of completing (neo)adjuvant trastuzumab (subgroup B; treatment must have been for ≥ 9 weeks), or progression within 6 months after initiation of first-line trastuzumab for advanced disease (subgroup C; treatment must have been for ≥ 6 weeks). A washout period of 4 weeks was required after last trastuzumab-based therapy. Patients with brain metastases that had been treated with local treatment or patients with stable brain metastases could be enrolled if they did not require dexamethasone or mannitol. The key exclusion criteria were meningeal and/or spinal cord metastases; other malignancies within 5 years, except for cured cervical cancer in situ, skin basal cell carcinoma, and skin squamous cell carcinoma; previous use of anti-HER2 TKI or antibody–drug conjugate with proven efficacy; active hepatitis B or C; history of transplantation; uncontrolled hypertension or diabetes mellitus; or lactating or pregnant women.

The study was conducted in accordance with the Declarations of Helsinki and Good Clinical Practice and was approved by the ethics committee of each participating center. Written informed consent was obtained from each patient. The study was registered with ClinicalTrials.gov, NCT04001621.

### Procedures

Patients received oral pyrotinib 400 mg once daily and oral capecitabine 1000 mg/m^2^ twice a day on days 1–14 of each 21-day cycle. Treatment was continued until disease progression, intolerable toxicity, or withdrawal of consent. Dose reductions, interruptions, and discontinuations of study drugs were allowed according to AEs. The dose of pyrotinib could be reduced stepwise from 400 to 320 mg to 240 mg. The dose of capecitabine was permitted to be reduced stepwise by 25%. Dose escalation was not permitted upon resolution of toxicity. The cumulative interruption time of pyrotinib should be no more than 14 days in each cycle; otherwise, patients would be withdrawn from the study.

Imaging examinations were performed every 6 weeks for the first 20 treatment cycles, and every 12 weeks thereafter. Radiological response was assessed by investigators according to RECIST 1.1. For patients who discontinued the study treatment before disease progression or death, subsequent imaging examinations were performed every 12 weeks until the initiation of other anti-cancer therapies, disease progression, or death. OS was followed every 12 weeks until loss to follow-up, death, or completion of the study. Adverse events (AEs) during the study treatment and until 28 days after the last dose of study drug were graded according to the National Cancer Institute Common Terminology Criteria for Adverse Events, version 4.03.

### Endpoints

The primary endpoint was PFS, defined as the time from the initiation of study treatment to the first disease progression per RECIST 1.1 or any-cause death, whichever came first.

Secondary endpoints included objective response rate (ORR; defined as the proportion of patients with the best response of complete response [CR] or partial response [PR] per RECIST 1.1), duration of response (defined as the time from the first CR or PR to disease progression per RECIST 1.1 in patients with confirmed objective response), disease control rate (DCR; defined as the proportion of patients with the best response of CR, PR, or stable disease per RECIST 1.1), overall survival (OS; defined as the time from the initiation of study treatment to any-cause death), and safety.

### Statistical analysis

Trastuzumab plus vinorelbine was the backbone treatment for trastuzumab-pretreated patients in 2019 in China; thus, the median PFS (5.78 months) of the control group in BOLERO-3 was considered as the historical control in our study [13]. The study treatment was expected to increase the median PFS to 8.0 months. Assuming that the survival time was in accordance with exponential distribution, 64 disease progression or death events were required to test the difference between the study treatment and historical control with a significance level of 5% and a power of 80%. The planned enrollment period was 32 months and the planned follow-up period was 24 months. Considering a dropout rate of 5%, 96 patients were required.

Efficacy and safety were analyzed in all patients with at least one dose of study drug. Continuous variables were expressed as median (range), and categorical variables were expressed as frequency (percentage). The 95% confidence intervals (CIs) of ORR and DCR were calculated using the Clopper-Pearson method. Comparison of ORR was performed between subgroups using the chi-square test. Median PFS and OS were estimated using the Kaplan–Meier method, and their 95% CIs were calculated using the Brookmeyer-Crowley method. Comparison of PFS was performed between subgroups using the Cox proportional hazard regression model. All statistical analyses were performed using SAS version 9.4 (SAS Institute, Cary, NC, USA). Two-sided *P* < 0.05 was considered statistically significant.

## Results

### Patient characteristics and treatment

**Fig. 1:**
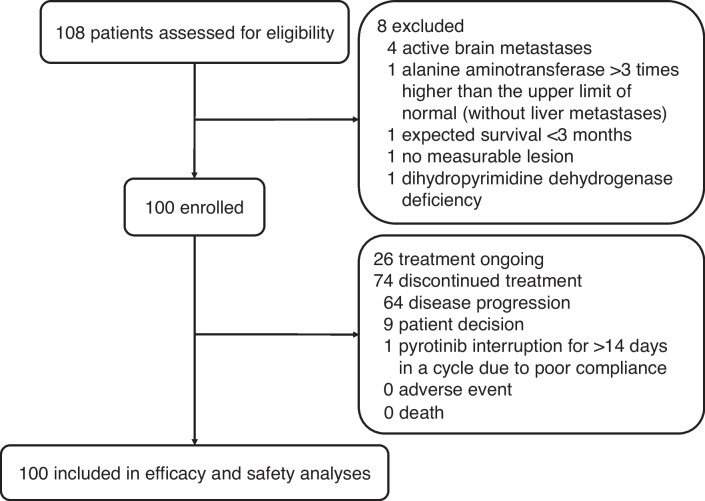
Patient flowchart

Between June 20, 2019, and September 19, 2021, a total of 108 patients were screened for eligibility, and 100 patients were enrolled and included in the efficacy and safety analyses (Fig. 1). Baseline characteristics are shown in Table 1. Of 100 patients, 65 (65.0%) had hormone receptor-negative disease, 94 (94.0%) had metastatic disease, 66 (66.0%) had visceral metastases, and 21 (21.0%) had prior use of pertuzumab. By the data cutoff date on August 31, 2022, the median follow-up duration was 20.1 months (range, 1.3–38.2). Median duration of the study treatment was 9.3 months (range, 0.2–38.2). Twenty-six (26.0%) patients were still on treatment.

**Table 1:** Patient characteristics

	**Patients (*n* = 100)**
**Age (years), median (range)**	51 (24–69)
< 65, *n* (%)	93 (93.0)
≥ 65, *n* (%)	7 (7.0)
**ECOG performance status, *n* (%)**	
0	29 (29.0)
1	71 (71.0)
**Hormone receptor status, *n* (%)**	
ER and/or PgR positive	35 (35.0)
ER and PgR negative	65 (65.0)
**Disease stage, *n* (%)**	
III	6 (6.0)
IV	94 (94.0)
**Visceral metastases, *n* (%)**	66 (66.0)
**Non-visceral metastases, *n* (%)**	34 (34.0)
**Site of metastases, *n* (%)**	
Brain	4 (4.0)
Lung	45 (45.0)
Liver	28 (28.0)
Bone	20 (20.0)
Lymph nodes	58 (58.0)
Others	45 (45.0)
**Primary trastuzumab resistance, *n* (%)**	
Progression during adjuvant trastuzumab	21 (21.0)
Progression within 12 months of completing adjuvant trastuzumab	49 (49.0)
Progression within 6 months after initiation of first-line trastuzumab for advanced disease	30 (30.0)
**Prior anti-HER2 therapy, *n* (%)**	
Trastuzumab	100 (100)
Pertuzumab	21 (21.0)
BAT8001	2 (2.0)
**Prior chemotherapy, *n* (%)**	
Taxane	96 (96.0)
Anthracycline	79 (79.0)
Cyclophosphamide	79 (79.0)
Platinum	22 (22.0)
Capecitabine	6 (6.0)
**Prior lines of chemotherapy for advanced disease, *n* (%)**	
0	66 (66.0)
1	32 (32.0)
2	2 (2.0)

*ECOG* Eastern Cooperative Oncology Group, *ER* estrogen receptor, *PgR* progesterone receptor, *HER2* human epidermal growth factor receptor 2

### Efficacy

**Fig. 2:**
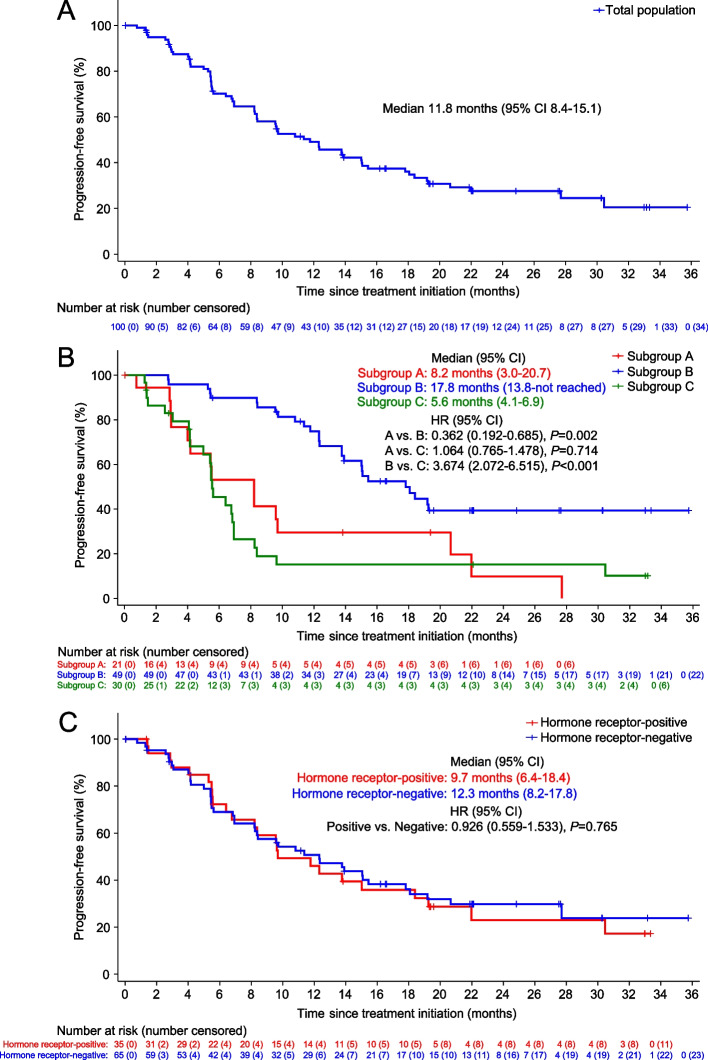
Kaplan–Meier curves for progression-free survival. **A** Total population. **B** Subgroup by the subcategory of primary trastuzumab resistance. Subgroup A included patients who had progression during adjuvant trastuzumab; subgroup B included patients who had progression within 12 months of completing adjuvant trastuzumab; subgroup C included patients who had progression within 6 months after the initiation of first-line trastuzumab for advanced disease. **C** Subgroup by hormone receptor status

By the data cutoff date, 66 (66.0%) of 100 patients had disease progression or died. The median PFS was 11.8 months (95% CI, 8.4–15.1; Fig. 2A). Twenty-five deaths occurred, and the median OS was not reached (95% CI, 29.0–not reached). The 1-year OS rate was 86.6%.

Further analyses showed that the median PFS was 8.2 months (95% CI, 3.0–20.7) in subgroup A (progression during adjuvant trastuzumab; *n* = 21), 17.8 months (95% CI, 13.8–not reached) in subgroup B (progression within 12 months of completing adjuvant trastuzumab; *n* = 49), and 5.6 months (95% CI, 4.1*–*6.9) in subgroup C (progression within 6 months after initiation of first-line trastuzumab for advanced disease; *n* = 30; Fig. 2B). No significant difference in median PFS was observed in subgroup by hormone receptor status (hormone receptor-positive: 9.7 months [95% CI, 6.4–18.4]; hormone receptor-negative: 12.3 months [95% CI, 8.2–17.8]; hazard ratio, 0.926 [95% CI, 0.559–1.533]; *P* = 0.765; Fig. 2C).

**Fig. 3:**
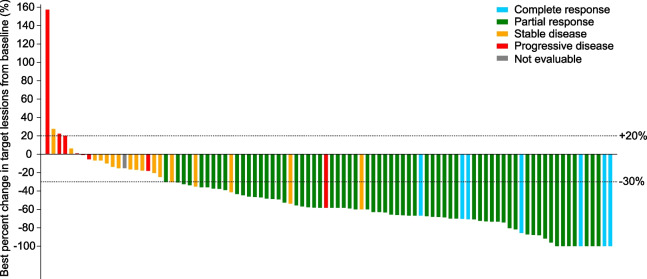
Waterfall plot for best percent change in target lesions from baseline among individual patients (*n* = 96). Four of 100 patients discontinued treatment before the first post-baseline imaging examination; their responses could not be assessed and were not shown in this figure. One of 96 patients had a stable disease and withdrew from the study due to personal reason without confirmation of response; the final response was deemed as not evaluable

Seven of 100 patients achieved confirmed CR and 63 achieved confirmed PR, with a confirmed ORR of 70.0% (95% CI, 60.0–78.8). Median duration of response was 13.8 months (95% CI, 10.2–19.3). Seven patients with CR were all from subgroup B. The ORR was significantly higher in subgroup B than in subgroups A and C (Additional file 1: Table S1), showing a similar trend with PFS. The DCR was 87.0% (95% CI, 78.8–92.9). Waterfall plot for best percent change in target lesions from baseline among individual patients is shown in Fig. 3.

### Safety

Treatment-emergent AEs (TEAEs) are summarized in Table 2. All patients had TEAEs, and 56 (56.0%) reported grade ≥ 3 TEAEs. The most common grade ≥ 3 TEAEs included diarrhea (24 [24.0%]), palmar-plantar erythrodysesthesia syndrome (nine [9.0%]), decreased neutrophil count (eight [8.0%]), hypokalemia (six [6.0%]), and anorexia (five [5.0%]). No grade 4 diarrhea occurred, while grade 3 diarrhea mostly (17/24, 70.8%) occurred in the first treatment cycle (Additional file 1: Fig. S1).

**Table 2:** Treatment-emergent adverse events

	Patients (*n* = 100)		
	**Grades 1–2**	**Grade 3**	**Grade 4**
Any event	44 (44.0)	53 (53.0)	3 (3.0)
Diarrhea	72 (72.0)	24 (24.0)	0
Anemia	52 (52.0)	4 (4.0)	0
Palmar-plantar erythrodysesthesia syndrome	46 (46.0)	9 (9.0)	0
White blood cell decreased	52 (52.0)	2 (2.0)	1 (1.0)
Neutrophil count decreased	35 (35.0)	7 (7.0)	1 (1.0)
Vomiting	37 (37.0)	2 (2.0)	0
Blood bilirubin increased	37 (37.0)	1 (1.0)	0
Creatinine increased	37 (37.0)	1 (1.0)	0
Aspartate aminotransferase increased	32 (32.0)	3 (3.0)	0
Alanine aminotransferase increased	31 (31.0)	3 (3.0)	0
Weight loss	34 (34.0)	0	0
Anorexia	27 (27.0)	5 (5.0)	0
Proteinuria	32 (32.0)	0	0
Hyperuricemia	31 (31.0)	0	0
Nausea	30 (30.0)	1 (1.0)	0
Urinary tract infection	29 (29.0)	0	0
Hypokalemia	21 (21.0)	5 (5.0)	1 (1.0)
Hypertriglyceridemia	23 (23.0)	0	1 (1.0)
Hematuria	19 (19.0)	0	0
Hypocalcemia	19 (19.0)	0	0
Stomatitis	19 (19.0)	0	0
Fatigue	17 (17.0)	0	0
Hypophosphatemia	16 (16.0)	1 (1.0)	0
Alkaline phosphatase increased	15 (15.0)	0	0
Hypoalbuminemia	14 (14.0)	0	0
Cardiac disorders	13 (13.0)	0	0
Platelet count decreased	12 (12.0)	1 (1.0)	0
Hypomagnesemia	12 (12.0)	0	0
Hyperglycemia	10 (10.0)	1 (1.0)	0
Sinus tachycardia	10 (10.0)	0	0
Upper respiratory tract infection	6 (6.0)	1 (1.0)	0
Rash	5 (5.0)	1 (1.0)	0
Gamma-glutamyltransferase increased	2 (2.0)	1 (1.0)	0
Lymphocyte count decreased	2 (2.0)	1 (1.0)	0
Fever	1 (1.0)	1 (1.0)	0
Pneumonitis	1 (1.0)	1 (1.0)	0
Blood chloride decreased	0	1 (1.0)	0
Electrocardiogram QT corrected interval prolonged	0	1 (1.0)	0
Fracture	0	1 (1.0)	0
Hypertension	0	1 (1.0)	0
Hyponatremia	0	1 (1.0)	0
Pericardial effusion	0	0	1 (1.0)
Pleural effusion	0	1 (1.0)	0
Thrombosis	0	1 (1.0)	0

Data are *n* (%). Grade 1–2 treatment-emergent adverse events occurring in at least 10% of patients and all grade 3 and 4 events are reported. Each patient was counted once for the highest grade of each event experienced. No treatment-related deaths occurred

Twelve (12.0%) of 100 patients had dose reductions of pyrotinib due to AEs, and 37 (37.0%) had dose reductions of capecitabine. Four (4.0%) patients discontinued capecitabine due to increased blood bilirubin, anemia, dyspepsia, and palmar-plantar erythrodysesthesia syndrome, respectively. No AEs leading to discontinuation of pyrotinib occurred. No treatment-related deaths occurred.

## Discussion

To our knowledge, this phase 2 trial was the first positive multicenter study for patients with HER2-positive advanced breast cancer and primary trastuzumab resistance, in contrast to previous failed trials in this clinical setting. The median PFS of 11.8 months met the primary endpoint, significantly longer than the pre-trial hypothesis of 8.0 months in the trial protocol. The combination of pyrotinib and capecitabine was also demonstrated well-tolerated and had no new safety signals in this study.

Patients with primary trastuzumab resistance are under-represented in two pivotal phase 3 trials (EMILIA and DESTINY-Breast 03). Even for those who received real second-line treatment in these two trials, they were either primary- or secondary-resistant to trastuzumab. The EMILIA study confirmed the role of T-DM1 in patients with HER2-positive advanced breast cancer previously treated with trastuzumab and a taxane when compared with lapatinib plus capecitabine (PFS: 9.6 vs. 6.4 months; hazard ratio, 0.65; *P* < 0.001; OS: 30.9 months vs. 25.1 months; hazard ratio, 0.68; *P* < 0.001) [19]. T-DXd defeated T-DM1 based on the amazing results from the phase 3 DESTINY-Breast 03 study (PFS: 28.8 months vs. 6.8 months; hazard ratio, 0.33; *P* < 0.0001) [38]. Both of these two studies included a subset of primary trastuzumab-resistant patients, mostly in the context of advanced disease, but the specific number of these patients and corresponding efficacy of T-DXd and T-DM1 are not available. While the whole world rejoices in the emergence of T-DXd, the hard-to-treat, primarily trastuzumab-resistant population still needs attention.

Among previous clinical trials focusing on trastuzumab-resistant patients, the definition of primary trastuzumab resistance is not unified, leading to hard indirect comparisons across studies (Additional file 1: Table S2) [11–18]. This definition in our study was generally consistent with that in the phase 3 LUX-Breast 1 study [14]. We have to acknowledge that the definition used in this trial is still arbitrary; a thorough research on baseline re-biopsy sample might be more informative. In LUX-Breast 1, the ORR and median PFS were 46.1% and 5.5 months with afatinib plus vinorelbine [14], respectively. Compared with these results, pyrotinib plus capecitabine in our study had a higher ORR (70.0%) and doubled the median PFS (11.8 months), demonstrating its high potency. Randomized controlled trials are warranted to validate the role of pyrotinib plus capecitabine in patients with primarily trastuzumab-resistant, HER2-positive advanced breast cancer and under the unified definition of primary trastuzumab resistance.

Patients who had progression within 12 months of completing adjuvant trastuzumab (subgroup B in our study) are generally included in the trastuzumab-resistant population [12–14, 16]. Pyrotinib plus capecitabine resulted in a long median PFS in this subpopulation (17.8 months), which may be not inferior to reported in the first-line trials for advanced disease [39–43]. On the other hand, patients with rapid progression on trastuzumab during adjuvant therapy (subgroup A: 8.2 months) or for advanced disease (subgroup C: 5.6 months) only achieved modest PFS benefit with pyrotinib plus capecitabine, suggesting that different mechanisms of drug resistance might be involved. Of course, caution should be taken to interpret the data due to the limited subgroup sample size in our study, and confirmation of real differences in subpopulations might need separate investigations in statistically powered trials.

The safety profile of pyrotinib plus capecitabine was consistent with results from previous clinical trials [36, 44–46]. As expected, the most common grade ≥ 3 AE was diarrhea, which mainly occurred in the first treatment cycle and could be managed with dose reductions of pyrotinib and antidiarrheal agents. No diarrhea or other AEs resulted in discontinuation of pyrotinib.

This study has some limitations. First, there might be potential bias due to the single-arm design without control group. In fact, it is very hard to conduct randomized controlled trials since such patients were rare due to advance in early diagnosis and HER2-targeted therapy, and it took us 27 months to enroll 100 patients at 16 sites. In the future, direct comparison between our study treatment and modern treatment option, especially T-DXd, is urgently needed. Second, only Chinese patients were enrolled and the effect of pyrotinib plus capecitabine in other populations needs to be established. Third, the efficacy was assessed by investigators rather than independent review committee. Fourth, due to the small sample size, multivariable analyses were not performed to analyze the independent factors influencing the treatment response and survival. Finally, OS data are not mature yet, which will be reported in the future.

## Conclusions

Pyrotinib plus capecitabine can be considered to be a treatment option in HER2-positive advanced breast cancer patients who have shown primary resistance to trastuzumab. Even in the era of modern anti-HER2 treatments, this clinical setting warrants more investigations to meet unmet needs.

### Supplementary Information

**Additional file 1:** **Table S1. **Tumor response. **Table S2. **Indirect comparison across studies. **Figure S1. **The incidence of diarrhea in each treatment cycle.

## Data Availability

The datasets used and/or analyzed during the current study are available from the corresponding author on reasonable request.

## Abbreviations

AE: Adverse event
AKT: Protein kinase B
CI: Confidence interval
CR: Complete response
DCR: Disease control rate
EGFR: Epidermal growth factor receptor
HER2: Human epidermal growth factor receptor 2
MAPK: Mitogen-activated protein kinase
mTOR: Mammalian target of rapamycin
ORR: Objective response rate
OS: Overall survival
PFS: Progression-free survival
PI3K: Phosphatidylinositol 3-kinase
PR: Partial response
RECIST: Response Evaluation Criteria In Solid Tumors
T-DM1: Trastuzumab emtansine
T-DXd: Trastuzumab deruxtecan
TEAE: Treatment-emergent adverse event
TKI: Tyrosine kinase inhibitor
ULN: Upper limit of normal
