# Supplementary material for: Pyrotinib plus capecitabine for trastuzumab-resistant, HER2-positive advanced breast cancer (PICTURE): a single-arm, multicenter phase 2 trial
Source: BMC Med. 2023 Aug 9;21:300. doi: 10.1186/s12916-023-02999-0 (PMC10410905; doi:10.1186/s12916-023-02999-0)
Supplement: Supplementary file 1 — Additional file 1: Table S1. Tumor response. Table S2. Indirect comparison across studies. Figure S1. The incidence of diarrhea in each treatment cycle. [file 12916_2023_2999_MOESM1_ESM.pdf]

Table S1. Tumor response

	Total (n=100)	Subgroup A (n=21)	Subgroup B (n=49)	Subgroup C (n=30)
**Confirmed response, n (%)**				
Complete response	7 (7.0)	0	7 (14.3)	0
Partial response	63 (63.0)	11 (52.4)	36 (73.5)	16 (53.3)
Stable disease	17 (17.0)	3 (14.3)	5 (10.2)	9 (30.0)
Progressive disease	8 (8.0)	3 (14.3)	1 (2.0)	4 (13.3)
Not evaluable	1 (3.3)	1 (3.3)	0	0
Not assessed*	4 (13.3)	3 (14.3)	0	1 (3.3)
**Objective response rate, n (%)**	70 (70.0)	11 (52.4)	43 (87.8)	16 (53.3)
95% confidence interval	60.0-78.8	29.8-74.3	75.2-95.4	34.3-71.7
Nominal *P* vs. subgroup A			0.001	0.947
Nominal *P* vs. subgroup C			<0.001	

Subgroup A included patients who had progression during adjuvant trastuzumab; subgroup B included patients who had progression within 12 months of completing adjuvant trastuzumab; subgroup C included patients who had progression within 6 months after the initiation of first-line trastuzumab for advanced disease.

*Four patients discontinued treatment before the first post-baseline imaging examination.

Table S2. Indirect comparison across studies

Study	Definition of primary trastuzumab resistance	Treatment	ORR	mPFS (months)
Morrow et al.	Have PD after ≥1 trastuzumab-based therapy for MBC	Everolimus + trastuzumab	NA	4.1 (n=47)
Hurvitz et al.	Progressed during or within 3 months of receiving the last dose of trastuzumab for advanced disease or recurred within 12 months of completing trastuzumab-based (neo)adjuvant therapy	Everolimus + trastuzumab + paclitaxel	22% (12/55)	5.5
BOLERO-3	Recurrence during or within 12 months of adjuvant treatment or progression during or within 4 weeks of treatment for advanced disease	Everolimus + trastuzumab + vinorelbine vs. Trastuzumab + vinorelbine	41% (116/284) vs. 37% (106/285)	7.0 vs. 5.8
LUX-Breast 1	Progressed on or within 12 months of completing adjuvant trastuzumab (treatment must have been for ≥9 weeks); progressed on or within 6 months of completing first-line trastuzumab for metastatic disease (treatment must have been for ≥6 weeks)	Afatinib + vinorelbine vs. Trastuzumab + vinorelbine	46% (154/334) vs. 47% (79/168)	5.5 vs. 5.6
PIKHER2	Trastuzumab-resistant	Buparlisib + lapatinib	4% (1/24)	NA
Pistilli et al.	Recurrence while on or within 4 weeks since the most	Buparlisib + trastuzumab	10% (5/50)	NA

	recent infusion for patients who received trastuzumab for metastatic disease (or within 12 months for patients who received trastuzumab as adjuvant therapy)			
Jain et al.	Locally advanced disease must be inoperable and have progressed within 6 months of trastuzumab and/or taxane treatment and metastatic disease must have progressed during or after trastuzumab and taxane therapy.	Alpelisib + T-DM1	43% (6/14)	8.1
PANACEA	Documented progression during previous trastuzumab-based therapy	Pembrolizumab + trastuzumab	PD-L1-positive: 15% (6/40) PD-L1-negative: 0% (0/12)	PD-L1-positive: 2.7 PD-L1-negative: 2.5
Present study (PICTURE)	Progression during (neo)adjuvant trastuzumab or within 12 months of completing (neo)adjuvant trastuzumab (treatment must have been for ≥9 weeks), or progression within 6 months after initiation of first-	Pyrotinib + capecitabine	70.0% (70/100)	11.8

	line trastuzumab for advanced disease (treatment must have been for ≥6 weeks)	

ORR, objective response rate; mPFS, median progression-free survival; NA, not applicable; T-DM1, trastuzumab emtansine; PD-L1, programmed cell death-ligand 1.

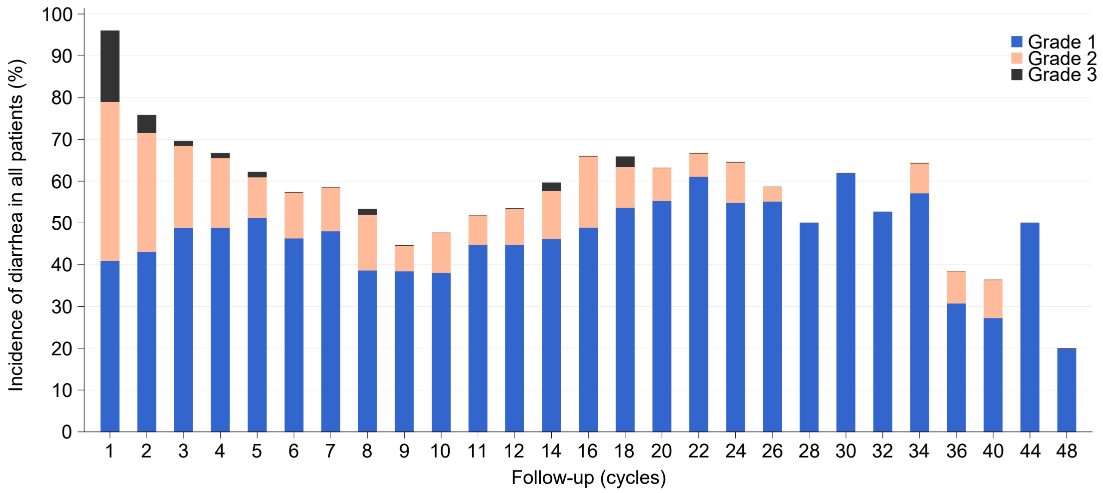

Grade 1	41	41	45	44	42	38	37	29	25	24	26	26	24	23	22	21	22	17	16	13	13	10	8	4	3	4	1
Grade 2	38	27	18	15	8	9	8	10	4	6	4	5	6	8	4	3	2	3	1	0	0	0	1	1	1	0	0
Grade 3	17	4	1	1	1	0	0	1	0	0	0	0	1	0	1	0	0	0	0	0	0	0	0	0	0	0	0
No. at risk	100	95	92	90	82	82	77	75	65	63	58	58	52	47	41	38	36	31	29	26	21	19	14	13	11	8	5

Fig. S1 The incidence of diarrhea in each treatment cycle.
